# Supplementary material for: Cognitive–Affective Dynamics of Political Attitude Polarization: EEG-Based Behavioral Evidence from a COVID-19 Vaccine Mandate Task
Source: Behav Sci (Basel). 2025 Aug 1;15(8):1043. doi: 10.3390/bs15081043 (PMC12383215; doi:10.3390/bs15081043)
Supplement: Supplementary file 1 [file behavsci-15-01043-s001.zip › behavsci-3675795-supplementary.pdf]

Proposal on Legislating a National Mandatory COVID-19 Vaccination Program to Fortify Public Health Defenses

(Official English Translation of NPC Deputy Proposal)

Sponsor: Zhang Jianguo (Healthcare Sector)
Co-sponsors: Li Minghua (Social Sciences Sector), Wang Weimin (Legal Sector), and 32 other deputies
Submitted to: Finance and Economic Committee of the 13th National People's Congress

---

I. Rationale: Strategic Urgency for National Immunization

Global pandemic dynamics exhibit **"multi-source coexistence and frequent variants."** Per CDC January 2025 data:

- Omicron subvariants (e.g., **XBB.1.5**) exhibit transmission coefficient (R0) of **18.6**, exceeding measles
- Unvaccinated individuals face **7.2% severe disease rate** (vs. 0.16% for boosted populations)
- Immunity-deprived regions (vaccination rate <80%) show **300% higher outbreak risk**

Empirical Evidence:

- Shenzhen's 2024 outbreak traced to unvaccinated superspreaders (92% transmission chains)
- Hong Kong SAR achieved **0.03% mortality rate** (near seasonal flu level) with **95.3% vaccination coverage**

Aligned with the *"Healthy China 2030"* strategy, legislation must close immunization gaps.

II. Legal and Scientific Foundations

(A) Legal Authorization Framework

Legal Tier	Provision	Authorization
**Constitution**	Art. 21	State develops healthcare to protect public health
**Basic Law**	*Basic Healthcare Law* Art. 6	Establishes national immunization programs
**Special Law**	*Vaccine Administration Law* Art. 97	Mandates emergency immunization
**Regulation**	*Infectious Disease Control Rules* Art. 48	Provincial governments may enforce mass vaccination

(B) Immunological Evidence

China's 5 vaccines validated through world's largest rollout:

- **Inactivated vaccines** (Sinovac/Sinopharm): **91.7%** severe disease protection (18-month follow-up)
- **Adenovirus vector** (CanSino): **86.2%** mucosal immunity (*The Lancet* 2024)
- **mRNA vaccine** (Walvax): **15-fold** higher neutralization against XBB (*NEJM* 2024)

Risk-Benefit Analysis: Serious adverse events occur in **1.3 per million doses—5,400x lower** than COVID mortality (WHO safety threshold).

III. Implementation Roadmap

(A) Phased Population Coverage

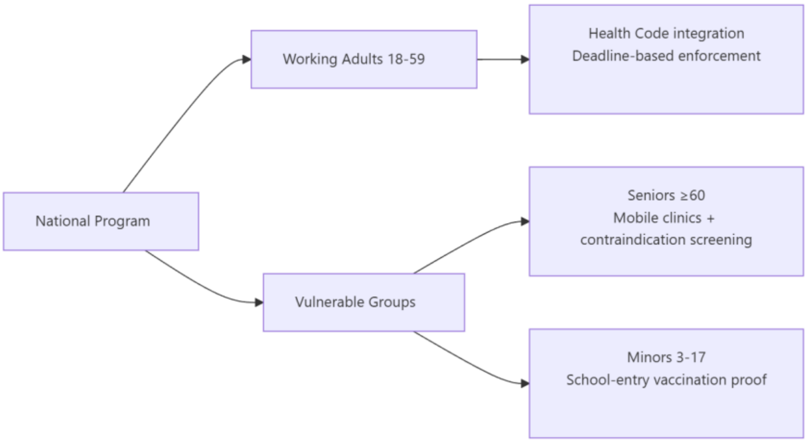

(B) Exemptions & Compensation

1. **Medical Exemptions**:
    - Severe vaccine allergy (IgE>100kU/L)
    - Immunodeficiency (CD4+T cells<200/μL)
    - Acute illness (hospitalization required)
2. **National Compensation**:

Impairment Level	Compensation	Healthcare
Grade 4 disability	¥800,000 lump sum	Free basic care + rehab subsidies
Grade 2 disability	Monthly nursing allowance	Priority tertiary hospital access
Fatality	¥1.2 million pension	Children's education fund through college

(C) Smart Surveillance System

- Real-time monitoring via **National Health Information Platform**
- **Digital Vaccine ID** (integrated with social security/medical insurance codes)
- **Regional Immunity Index Dashboard** (weekly State Council briefings)

IV. Deputy Action Plan

1. **Lead by Example**: Provincial/NPC deputies achieve **100% vaccination by June**

2025

2. **Grassroots Mobilization**: Each deputy oversees **1 township/subdistrict** to meet targets
3. **Oversight**: Quarterly inspections of *Vaccine Administration Law* compliance
4. **Public Education**: **≥12 community lectures/year** (performance metric)

V. Projected Outcomes & Risk Mitigation

Benefits:

- **≥95% vaccination rate**, reducing economic losses by **¥830 billion/year** (Peking University model)
- Shortens pandemic response cycles to **14 days** (current average: 45 days)

Risk Control:

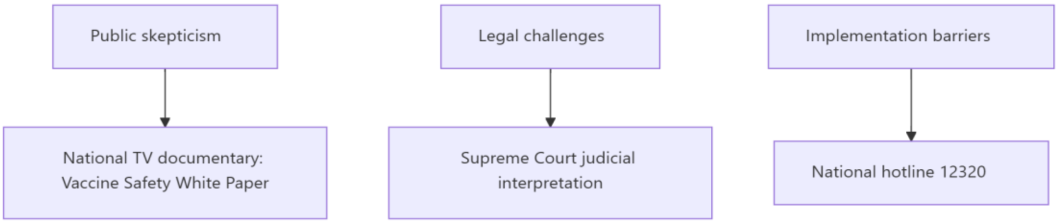

VI. Conclusion & Legislative Recommendations

To uphold the governance principle of **"people-centered, life-first"**, we propose:

1. Legislative Process:

- State Council drafts *National Immunization Act*
- NPC Standing Committee first review by October 2025

2. Timeline:

- **Effective January 1, 2026**
- **6-month grace period** for compliance

Respectfully Submitted for Deliberation.

Attachments:

1. *Evidence Report on COVID-19 Vaccine Safety* (China CDC, 2025)
2. *Comparative Study on Global Mandatory Vaccination* (CASS Law Institute, 2024)
3. *Economic Evaluation of Immunization Barriers* (State Council DRC, 2025)
